# Characterization of a d-lyxose isomerase from *Bacillus velezensis* and its application for the production of d-mannose and l-ribose

**DOI:** 10.1186/s13568-019-0877-3

**Published:** 2019-09-16

**Authors:** Zongren Guo, Liangkun Long, Shaojun Ding

**Affiliations:** 1 The Co-Innovation Center of Efficient Processing and Utilization of Forest Resources, Nanjing Forestry University, Nanjing, 210037 China; 2 Jiangsu Key Lab for the Chemistry & Utilization of Agricultural and Forest Biomass, Nanjing Forestry University, Nanjing, 210037 China; 3 College of Chemical Engineering, Nanjing Forestry University, Nanjing, 210037 China

**Keywords:** d-Mannose, l-Ribose, l-Arabinose isomerase, d-Lyxose isomerase, *Bacillus velezensis*

## Abstract

d-Mannose and l-ribose are two important monosaccharides, which have attracted public attention recently because of their great application potentials in food, cosmetic and pharmaceutical industries. Sugar isomerases catalyze the sugar isomerization and therefore can be used as the biocatalysts for production of the high-value sugars from inexpensive sugars. l-arabinose isomerase catalyzes the conversion of l-arabinose to l-ribulose, while d-lyxose isomerase catalyzes l-ribulose and d-fructose to l-ribose and d-mannose, respectively. In this paper, a putative d-LI from *Bacillus velezensis* (BvLI) was identified, characterized and used to produce d-mannose and l-ribose from d-fructose and l-arabinose, respectively. The recombinant BvLI exhibited a maximum activity at 55 °C and pH 6.5, in the presence of 0.1 mM Co^2+^. Approximately 110.75 g/L d-mannose was obtained from 500 g/L d-fructose in 6 h by the recombinant BvLI, and approximately 105 g/L l-ribose was obtained from 500 g/L l-arabinose in 8 h by the successive biocatalysis of l-arabinose isomerase from *Bacillus licheniformis* (BlAI) and BvLI.

## Introduction

l-Ribose is the C-2 epimer of l-arabinose and the aldose isomer of l-ribulose. l-ribose can be used as initial material to synthesize various l-nucleoside derivatives as anticancer and antiviral drugs (Wang et al. 1998; Okano 2009; Xu et al. 2016). d-Mannose is the C-2 aldose isomer of d-fructose and is a functional monosaccharide. It can promote the growth of intestinal probiotics and is used for the synthesis of the anti-tumor and immunostimulating drugs (Korneeva et al. 2012; Ranta et al. 2012; Zhang et al. 2017). Therefore, the synthesis of l-ribose and d-mannose was urgently demanded in industry (Beerens et al. 2012; Ban et al. 2017).

Many high-value monosaccharides were obtained from their isomeric forms by enzymatic reaction using sugar isomerases according to Izumoring strategy (Izumori 2006). Sugar isomerases possess diverse substrate specificities; therefore can be applied to produce many high-value sugars from their abundant and inexpensive counterparts in nature (Patel et al. 2011; Beerens et al. 2012). d-Lyxose isomerase (d-LI, EC 5.3.1.15) is a crucial enzyme involves in microbial pentose metabolism (Cho et al. 2007; Kobayashi et al. 2018). It displayed extensive substrate specificity on aldose-ketose, and ability to catalyze the isomerization reaction between d-lyxose and d-xylulose, d-mannose and d-fructose, as well as l-ribose and l-ribulose, respectively. Recently, several d-lyxose isomerases have been identified and characterized (Anderson and Allison 1965; Cho et al. 2007; Kwon et al. 2010; Park et al. 2010a; Van et al. 2010; Marles-Wright and Lewis 2011; Choi et al. 2012; Yu et al. 2016).

Due to the broad substrate specificity of d-LI, l-ribose and d-mannose can be produced by the d-LI-catalyzed isomerization of l-ribulose and d-fructose, respectively. However, l-ribulose is an expensive and scarce sugar, so it is uneconomical to synthesize l-ribose from l-ribulose. Previous researches demonstrated that l-ribulose could be obtained from l-arabinose by the isomerization catalysis of various l-arabinose isomerases (l-AI, EC 5.3.1.4) from *Bacillus licheniformis* (Zhang et al. 2010), *Bacillus coagulans* (Zhou and Wu 2012) and *Candida tropicalis* (Yeo et al. 2018). Therefore, in an alternative way, l-ribose can be obtained from l-arabinose, a cheap and widely available monosaccharide in lignocellulosic materials, via l-ribulose as an intermediate by a two-step isomerization using l-arabinose isomerase and d-lyxose isomerase.

In order to meet the demand for production of high-value sugars in industry, sugar isomerases need to have good biochemical properties and thermal stability (De et al. 2017). The isomerization reaction catalyzed by sugar isomerases should be performed in slightly acidic to reduce unwanted by-products (Rhimi et al. 2009; Nguyen et al. 2018). But until now, few d-LIs can completely satisfy the industrial requirement. Therefore, novel d-LIs still need to be identified and characterized.

In this study, we identified a putative d-lyxose isomerase from *B. velezensis* CICC 24777. The putative d-LI from *B. velezensis* CICC 24777 was characterized and evaluated for its potential in the application for production of d-mannose and l-ribose.

## Materials and methods

### Materials

All monosaccharides were purchased from Aladdin (Shanghai, China). FastPfu DNA polymerase and genomic DNA extraction kit were all procured from Transgene (Beijing, China). Restriction enzymes were all obtained from Takara (Japan). 2 × Hieff Clone Enzyme Premix, Ni–NTA agarose gel column and sodium dodecyl sulfate–polyacrylamide gel electrophoresis (SDS-PAGE) were obtained from Yeasen (Shanghai, China). The pET-21b (+) expression vector was obtained from Miaoling (Hangzhou, China). And all other chemicals and reagents were analytical grade from Sinopharm group (Beijing, China).

### Bacterial strains and culture conditions

*Bacillus velezensis* CICC 24777 strain was from this laboratory, and it was also deposited in China Center of Industrial Culture Collection (CICC). The host strains for gene cloning and expression were *E. coli* Top10 and *E. coli* BL21 (DE3) strains (Transgeen, Beijing, China), respectively. The *E. coli* cells were incubated in Luria-Bertani (LB) medium in a shaker (200 rpm, 37 °C).

### Cloning and expression of the BvLI

According to the whole genomic sequence of *B. velezensis* DSM 7 (NCBI accession number: NC 014551.1), a putative d-lyxose isomerase gene sequence (WP 013351044.1) was identified. Based on the gene sequence, two oligonucleotide primes (the restriction enzyme sites were underlined) were designed to clone BvLI gene: BvLI-F:5′-TAAGAAGGAGATATACATATGACGATATCGAAGCATGATGT-3′ (*Nde*I); BvLI-R:5′-TCAGTGGTGGTGGTGGTGGTGCTCGAGAATGCGCGGGTCGGTGAAAA-3′ (*Xho*I). Then the fragment encoding for the d-lyxose isomerase (BvLI) was amplified by PCR using the primers BvLI-F/BvLI-R and genomic DNA of *B. velezensis* CICC 24777 as template. The fragment was purified and inserted into pET-21b (+) digested with *Nde*I and *Xho*I by 2 × Hieff Clone Enzyme Premix. The pET21b-BvLI was transformed into *E. coli* Top10 cells and BvLI gene was verified by sequencing. Then, the pET21b-BvLI was again transformed into *E. coli* BL21 (DE3) cells. The recombinant cells were cultured in LB medium including 100 μg/mL ampicillin at 37 °C until to the appropriate optical density (OD_600_ = 0.5), then 0.5 mM isopropyl-β-d-thiogalactopyranoside (IPTG) was added and cells were cultured at 25 °C for 8 h.

### Purification of recombinant BvLI

The induced cells harboring pET21b-BvLI plasmid were collected by centrifugation (8000×*g*, 20 min). Then cell pellets were resuspended in lysis buffer (50 mM NaH_2_PO_4_, 250 mM NaCl, pH 8.0) and disrupted by sonication. Cell debris was precipitated after centrifugation (12,000×*g*, 20 min). The crude extract was loaded into a Ni–NTA agarose gel column previously equilibrated with the lysis buffer including 5 mM imidazole, and the enzyme was purified according to the manufacturer’s instructions. The purified enzyme solution was dialyzed against phosphate buffer (50 mM, pH 8.0) at 4 °C for 24 h. The protein concentration was assayed by BCA Protein Assay Kit (Sangon, Shanghai, China). The molecular mass of the recombinant enzyme was analyzed using 12% SDS-PAGE.

### Cloning, expression and purification of the recombinant BlAI

According to the previously reported sequence encoding for the l-arabinose isomerase (BlAI) from *B. licheniformis* (Prabhu et al. 2008), the gene (NCBI accession number: 3031327) was amplified from genomic DNA of *B. licheniformis* by using following primes (the restriction enzyme sites were underlined): BlAI-F:5′-TAAGAAGGAGATATACCATGGGCATGTTAACAACAGGGAAAAA-3′ (*Nco*I); BlAI-R: 5′-TCAGTGGTGGTGGTGGTGGTGCTCGAGCTTAATCACTACATATTCCA-3′ (*Xho*I). The amplified DNA fragment was inserted into pET-28a (+) digested with *Nco*I and *Xho*I. Then pET28a-BlAI was transformed into *E. coli* BL21 (DE3) cells, the cells were incubated in LB medium including 100 μg/mL kanamycin and induced by IPTG for 8 h at 25 °C. The recombinant BlAI was purified and analyzed according to the above method.

### Enzyme assay

The activity of the recombinant BvLI was assayed by determination of the amount of ketose obtained from the corresponding aldose. The reaction was proceeded in 50 mM sodium phosphate buffer (pH 6.5) including 10 mM l-ribose, 0.1 mM CoCl_2_ and 0.5 U/mL of enzyme at 55 °C for 30 min. Then the reaction was terminated by cooling samples on ice. The generated l-ribulose was measured by cysteine carbazole sulfuric acid method, and the absorbance was determined at 560 nm (Dische and Borenfreund 1951). One unit of the recombinant BvLI activity was defined as the amount of enzyme that formed 1 μmol l-ribulose from l-ribose per min at 55 °C and pH 6.5. The activity of the recombinant BlAI was determined according to the previous method described by Prabhu et al. (2008).

### Effects of temperature and pH on recombinant BvLI activity

The influence of temperature on the recombinant BvLI activity was measured by varying temperatures from 30 °C to 65 °C in pH 6.5. The influence of pH on the recombinant BvLI activity was measured at 55 °C with three different buffers, citrate buffer (50 mM pH 5.0–6.0), phosphate buffer (50 mM pH 6.0–8.0) and Tris–HCl buffer (8.0–9.0). The activity at each temperature or pH was relative to the maximum activity value (100%).

Thermostability of the recombinant BvLI was investigated by assaying the residual enzyme activity after preincubating enzyme at a range of 45 °C to 60 °C for a specific time. The pH stability of the recombinant BvLI was evaluated by preincubating enzyme in 50 mM phosphate buffer (pH 5.0–9.0) for 24 h. Then the enzyme activity was determined under standard reaction condition. The activity value of the recombinant BvLI without preincubation was determined as 100%.

### Effect of metal ions on the activity of recombinant BvLI

To determine the influence of metal ions on the recombinant BvLI activity, the recombinant BvLI was first dialyzed against pH 8.0 including 10 mM ethylene diamine tetraacetic acid (EDTA) at 4 °C for 12 h, then enzyme was dialyzed against EDTA-free phosphate buffer (pH 8.0) at 4 °C for 12 h. The activity of the recombinant BvLI was determined at 55 °C and pH 6.5 containing several metal ions (Cu^2+^, Zn^2+^, Fe^3+^, Mg^2+^, Ca^2+^, Co^2+^, Mn^2+^ and Ni^2+^) at a concentration of 1 mM. The influences of Co^2+^ and Mn^2+^ concentration on the recombinant BvLI activity were also measured by adding Co^2+^ or Mn^2+^ at concentrations from 0.1 to 1.5 mM. The activity of the recombinant BvLI without adding metal ion was used as a control (100%).

### Determination of specific activity and kinetic parameters

The specific activity of the recombinant BvLI was assayed under the standard conditions by using 10 mM d-lyxose, d-mannose and l-ribose. Kinetic parameters of the recombinant BvLI were measured at 55 °C and pH 6.5 including 0.1 mM CoCl_2_ and 0 to 300 mM substrate. The kinetic parameters Michaelis–Menten constant (*K*_m_) and turnover number value (*K*_cat_) were measured by the Michaelis–Menten equation.

### Bioconversion of d-fructose to d-mannose by recombinant BvLI

The effect of loading of the recombinant BvLI on the production of d-mannose was first studied. The loading of enzyme was varied from 5 to 30 U/mL for d-mannose production, and the reaction mixture (100 mL) was conducted in pH 6.5 including 500 g/L d-fructose, 0.1 mM CoCl_2_ and at 55 °C for 8 h. Subsequently, the effect of substrate concentration on the conversion rate of d-mannose was also investigated. The reaction was performed in pH 6.5 containing 25 U/mL enzyme and at 55 °C for 8 h, and the initial concentrations of d-fructose were 100, 200, 300, 400 and 500 g/L, respectively. Finally, the d-mannose production was proceeded in pH 6.5 including 500 g/L d-fructose, 25 U/mL enzyme, and 0.1 mM CoCl_2_ at 55 °C for 8 h.

### l-Ribose synthesis from l-arabinose by recombinant BlAI and BvLI

Biotransformation of l-arabinose to l-ribose by using the recombinant BlAI and BvLI was proceeded in an orderly way. l-arabinose was first catalyzed by the recombinant BlAI (1 unit per gram of substrate) in pH 7.0 including 500 g/L l-arabinose, 0.1 mM CoCl_2_ and 1.0 mM MnCl_2_ at 50 °C for 1 h (equilibrium phase), then the second reaction was performed at 55 °C by adding the different amount of recombinant BvLI (0.5–3.0 units per gram of substrate) for optimization of the enzyme loading. To investigate the effect of extra BvLI on the l-ribose conversion, additional 2.5 U/g l-arabinose of BvLI was supplemented at 2 h at the second reaction and the final concentration of l-ribose was compared with that of 2.5 U/g l-arabinose of BvLI at 1 h only. Finally, the l-ribose conversion was systematically investigated at l-arabinose concentration ranged from 100 to 500 g/L to biosynthesize l-ribose using 1 U/g l-arabinose of BlAI at the first reaction and 2.5 U/g l-arabinose of BvLI at the second reaction.

### Analytical methods

The concentrations of monosaccharide were detected by high-performance liquid chromatography (HPLC) system (Agilent 1200 series, USA) with a refractive index detector (Shimadzu) and a Sugar-pak1 column (6.5 mm × 300 mm, Waters). The column was eluted at 80 °C with deionized water at a flow rate of 0.4 mL/min (Mei et al. 2016).

## Results

### Cloning, expression and purification of recombinant BvLI and BlAI

The putative BvLI gene (501 bp; GenBank accession number: MK836420) was successfully amplified from the genomic DNA of *B. velezensis* CICC 24777 and sub-cloned into pET-21b vector. The BvLI gene encodes 167 amino acids with theoretical molecular mass and isoelectric point values of 20042.28 Da and 5.11, respectively. The amino acid sequence of the putative BvLI exhibited high identity (77.84%) with d-LI from *B. licheniformis* (Table 1). In addition, the phylogenetic tree further revealed a near relationship of the putative BvLI with d-LI from *B. subtilis*, d-LI from *B. vallismortis* and d-LI from *B. licheniformis* d-LI as well (Additional file 1: Fig. S1). Alignment of the amino acid sequence of BvLI with various characterized d-LIs confirmed that the metal coordination sites (H69, H71, E82 and H137) and substrate binding residues (K56, K80, E149 and D156) in BvLI were also conserved as all other d-LIs (Additional file 1: Fig. S2).

**Table 1:** Comparison of enzymatic character and amino acid sequences of d-LIs from various microorganisms

Origins	Optimum temperature (°C)	Optimum pH	Optimum metal ion and concentration	Identity (%)^a^	References
*Cohnella laevoribosii* RI-39	70	6.5	Mn^2+^, 1 mM	55.49	Cho et al. (2007)
*Providencia stuartii* KCTC 2568	45	7.5	Mn^2+^, 1 mM	48.63	Kwon et al. (2010)
*Serratia proteamaculans* KCTC 2936	40	7.5	Mn^2+^, 1 mM	17.11	Park et al. (2010a)
*Escherichia coli* O157:H7	50	7.5	Mn^2+^, 1 mM	18.06	Van et al. (2010)
*Bacillus licheniformis* DSM 13	40–45	7.5–8.0	Mn^2+^, 1 mM	77.84	Patel et al. (2011)
*Dictyoglomus turgidum* DSM 6724	75	7.5	Co^2+^, 0.5 mM	55.80	Choi et al. (2012)
*Thermosedimini*-*bacter oceani* DSM 16646	65	6.5	Mn^2+^, 1 mM	61.88	Yu et al. (2016)
*Bacillus velezensis* CICC 24777	55	6.5	Co^2+^, 0.1 mM	100	This study

^a^The identity of amino acid sequences between recombinant BvLI and other reported d-LIs

**Fig. 1:**
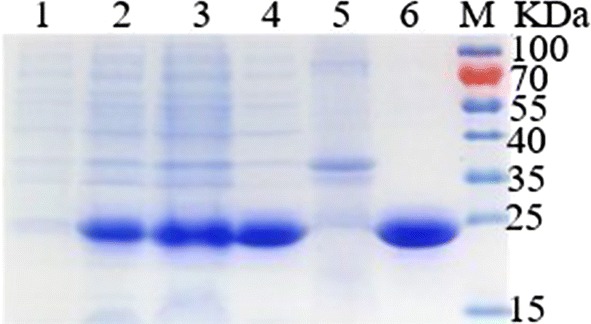
SDS-PAGE analysis of purified recombinant BvLI. Lane 1–3: whole *E. coli* BL21 (DE3) cells without IPTG induction, after IPTG induction for 4 h and 8 h, respectively; Lane 4, 5: supernatant and debris of induced cell lysate; Lane 6: purified recombinant BvLI; Lane M: protein marker

After recombinant *E. coli* BL21 (DE3) cells were induced with 0.5 mM IPTG at 25 °C for 8 h, the target protein was purified via affinity chromatography with Ni–NTA agarose gel and subsequently analyzed by 12% SDS-PAGE. According to SDS-PAGE (Fig. 1), the supernatant contained a strong band of the target protein, while the cell debris contained little target protein. The molecular mass of purified target protein was approximately 20 kDa, in accordance with the theoretical molecular weight. Which indicated that BvLI has been overexpressed as a soluble form protein in *E. coli* BL21 (DE3) cells.

The recombinant BlAI was also purified and analyzed according to the above method, the molecular mass of purified recombinant BlAI was approximately 53 kDa (Additional file 1: Fig. S3), it was in line with the reported molecular weight of recombinant BlAI.

### The effects of temperature and pH on the activity of recombinant BvLI

**Fig. 2:**
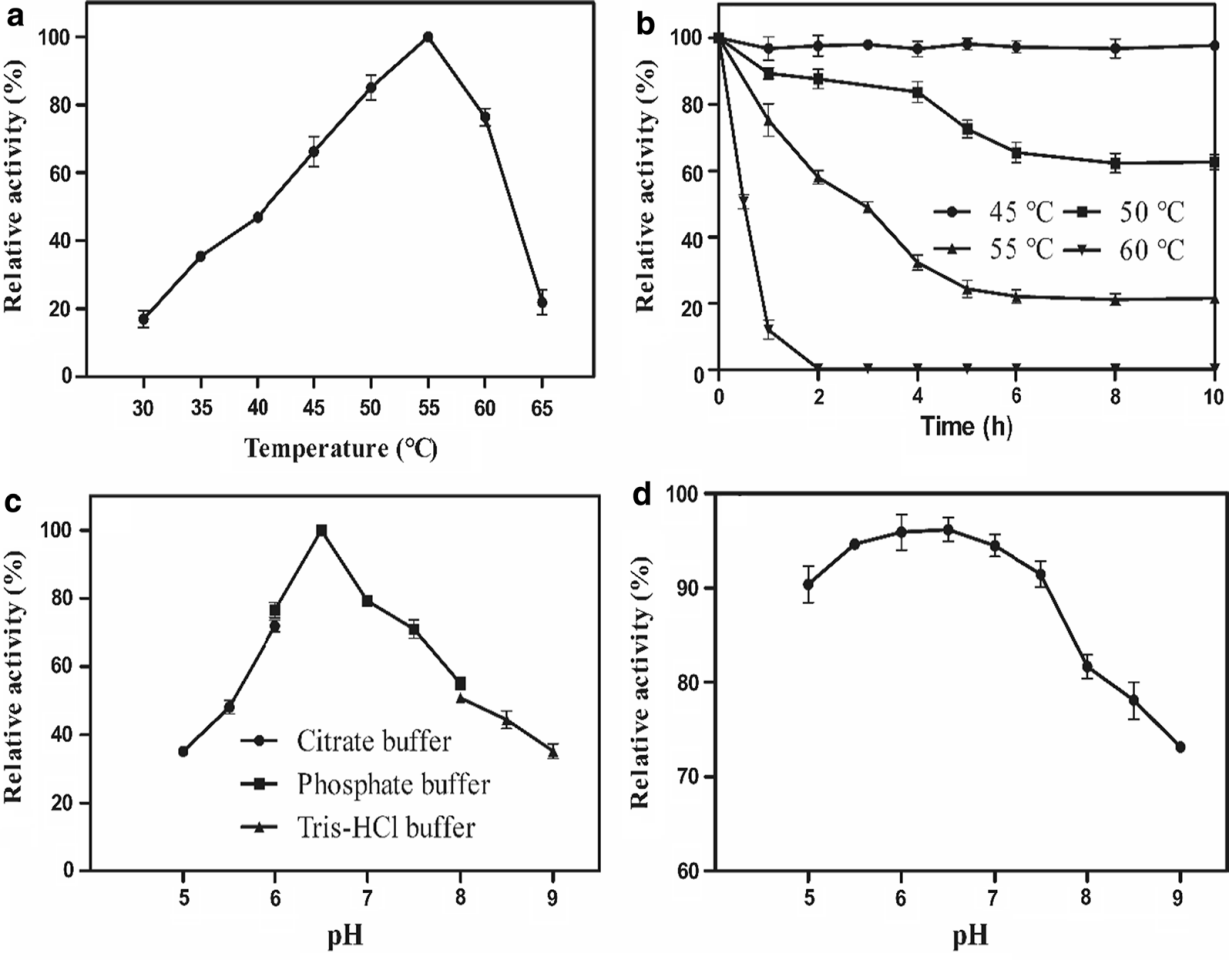
The effects of temperature and pH on the activity and stability of recombinant BvLI. **a** The effect of temperature on the recombinant BvLI activity; **b** the effect of temperature on the thermostability of recombinant BvLI. **c** The effect of pH on the recombinant BvLI activity. **d** The effect of pH on the stability of recombinant BvLI. Data represented the mean ± standard deviation from triplicate experiments

The influences of temperature on the recombinant BvLI activity and stability were determined and displayed in Fig. 2a, b. The optimal temperature of the recombinant BvLI was observed at 55 °C, and the enzyme remained over 60% relative activity at 45 °C to 60 °C. The enzyme activity decreased sharply when the temperature exceeded 60 °C, and only approximately 20% of initial activity was detected at 65 °C (Fig. 2a). BvLI is stable at temperature below 50 °C, and over 90% and 60% of the initial activities were remained after incubating the enzyme at 45 °C and 50 °C for 10 h, respectively. Approximately 21% of the initial activity were still retained after incubating the enzyme at 55 °C for 10 h and the half-life time is 3 h at 55 °C. The thermostability was decreased rapidly, and the half-life time was only about 0.5 h at 60 °C (Fig. 2b).

The recombinant BvLI displayed the highest activity at pH 6.5 and retained over 70% of the initial activity at pH 6.0–7.5 (Fig. 2c). As shown in Fig. 2d, the enzyme remained over 90% of the initial activity at pH 5.0–7.5 and over 70% of the initial activity at pH 7.5–9.0 after incubating recombinant BvLI in 50 mM phosphate buffer for 24 h, respectively.

### The effect of metal ions on the activity of recombinant BvLI

**Fig. 3:**
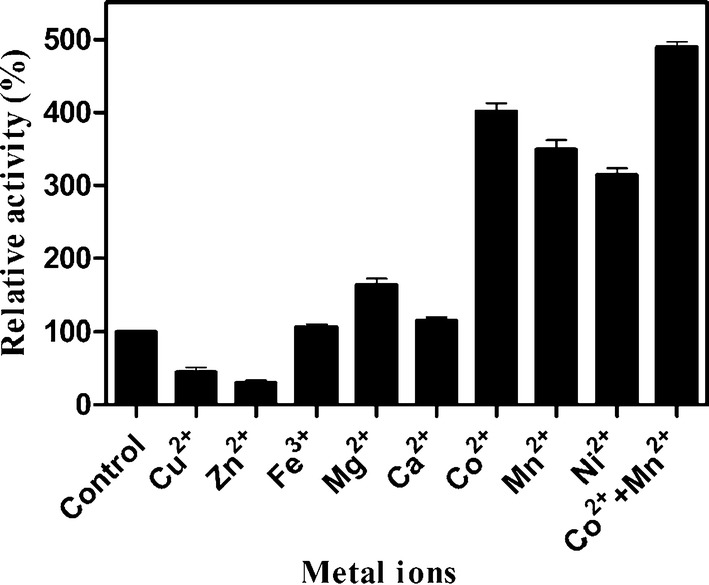
The effect of metal ions on the activity of recombinant BvLI. Data represented the mean ± standard deviation from triplicate experiments

The activity of the recombinant BvLI was detected at 55 °C and pH 6.5 containing various 1.0 mM metal ions. As displayed in Fig. 3, Co^2+^, Mn^2+^, Ni^2+^ and Mg^2+^ significantly increased the enzyme activity approximately 4.1-, 3.6-, 3.0- and 1.6-fold, respectively. Fe^3+^ and Ca^2+^ have little effect on the enzyme activity, while Cu^2+^ and Zn^2+^ intensively inhibited the enzyme activity. The optimal concentrations of Co^2+^ and Mn^2+^ to stimulate the enzyme activity were 0.1 and 1.0 mM, respectively (Additional file 1: Fig. S4). Since Co^2+^ and Mn^2+^ can each stimulate the catalytic activity of recombinant BvLI, the combined effect of both metals on the activity of the recombinant BvLI was also assayed in this study. The enzyme activity was improved approximately 4.9-fold when simultaneously adding 0.1 mM Co^2+^ and 1.0 mM Mn^2+^ (Fig. 3).

### Specific activity and kinetic parameters of recombinant BvLI

The specific activity and kinetic constant of the recombinant BvLI for d-lyxose, d-mannose and l-ribose were shown in Table 2. The specific activity of the recombinant BvLI for d-lyxose was 3.38- and 4.40-fold higher than d-mannose and l-ribose, respectively. The *K*_m_ of the recombinant BvLI for d-lyxose was lower than d-mannose and l-ribose, which revealed that the recombinant BvLI exhibited the highest affinity toward d-lyxose. In addition, the *K*_cat_ and *K*_cat_/*K*_m_ of the recombinant BvLI for d-lyxose were also higher than d-mannose and l-ribose. These results demonstrated that d-lyxose was the most favorable substrate for the recombinant BvLI.

**Table 2:** Specific activity and Kinetic parameters of recombinant BvLI^a^

Substrate	Specific activity (μmol/min/mg)	*K*_m_ (mM)	*K*_cat_ (min^−1^)	*K*_cat_/*K*_m_ (min/mM)
d-Lyxose	2.20 ± 0.12	33.14 ± 2.53	1335.89 ± 22.54	40.29 ± 0.38
d-Mannose	0.65 ± 0.04	55.80 ± 5.68	1213.46 ± 34.59	21.78 ± 0.91
l-Ribose	0.50 ± 0.06	151.4 ± 16.33	975.45 ± 50.13	6.44 ± 0.11

^a^Values represented the mean ± standard deviation from triplicate experiments

However, the ratio of *K*_cat_/*K*_m_ value of d-mannose or l-ribose to d-lyxose, reflecting the relative specificity bias among three sugars, was different among d-LIs (Table 3). The ratio *K*_cat_/*K*_m_ value of the recombinant BvLI for l-ribose to d-lyxose was 66.58-fold higher than d-LI from *Cohnella laevoribosii* RI-39, and the *K*_cat_/*K*_m_ ratio of the recombinant BvLI for d-mannose to d-lyxose was higher among reported d-LIs, except of d-LI from *Thermosediminibacter oceani* DSM 16646 and *E. coli* O157:H7. These results suggested that the recombinant BvLI has higher relative substrate specificity toward d-mannose and l-ribose compared with several reported d-LIs.

**Table 3:** The *K*_cat_/*K*_m_ ratio of recombinant d-LIs for d-mannose or l-ribose to d-lyxose

Origins	(*K*_cat_/*K*_m_)_d-mannose_/(*K*_cat_/*K*_m_)_d-lyxose_	(*K*_cat_/*K*_m_)_l-ribose_/(*K*_cat_/*K*_m_)_d-lyxose_	References
*C. laevoribosii* RI-39	0.0165	0.0024	Cho et al. (2007)
*P. stuartii* KCTC 2568	0.1734	NR	Kwon et al. (2010)
*S. proteamaculans* KCTC 2936	0.2152	NR	Park et al. (2010a)
*E. coli* O157:H7	0.7529	NR	Van et al. (2010)
*B. licheniformis*	0.5000	NR	Patel et al. (2011)
*D. turgidum* DSM 6724	0.1538	NR	Choi et al. (2012)
*T. oceani* DSM 16646	0.8607	NR	Yu et al. (2016)
*B. velezensis* CICC 24777	0.5406	0.1598	This study

(*K*_cat_/*K*_m_)_d-mannose_ *K*_cat_/*K*_m_ of recombinant d-LIs for d-mannose

(*K*_cat_/*K*_m_)_l-ribose_ *K*_cat_/*K*_m_ of recombinant d-LIs for l-ribose

(*K*_cat_/K_m_)_d-lyxose_ *K*_cat_/*K*_m_ of recombinant d-LIs for d-lyxose

*NR* not reported

### d-Mannose production from d-fructose by recombinant BvLI

**Fig. 4:**
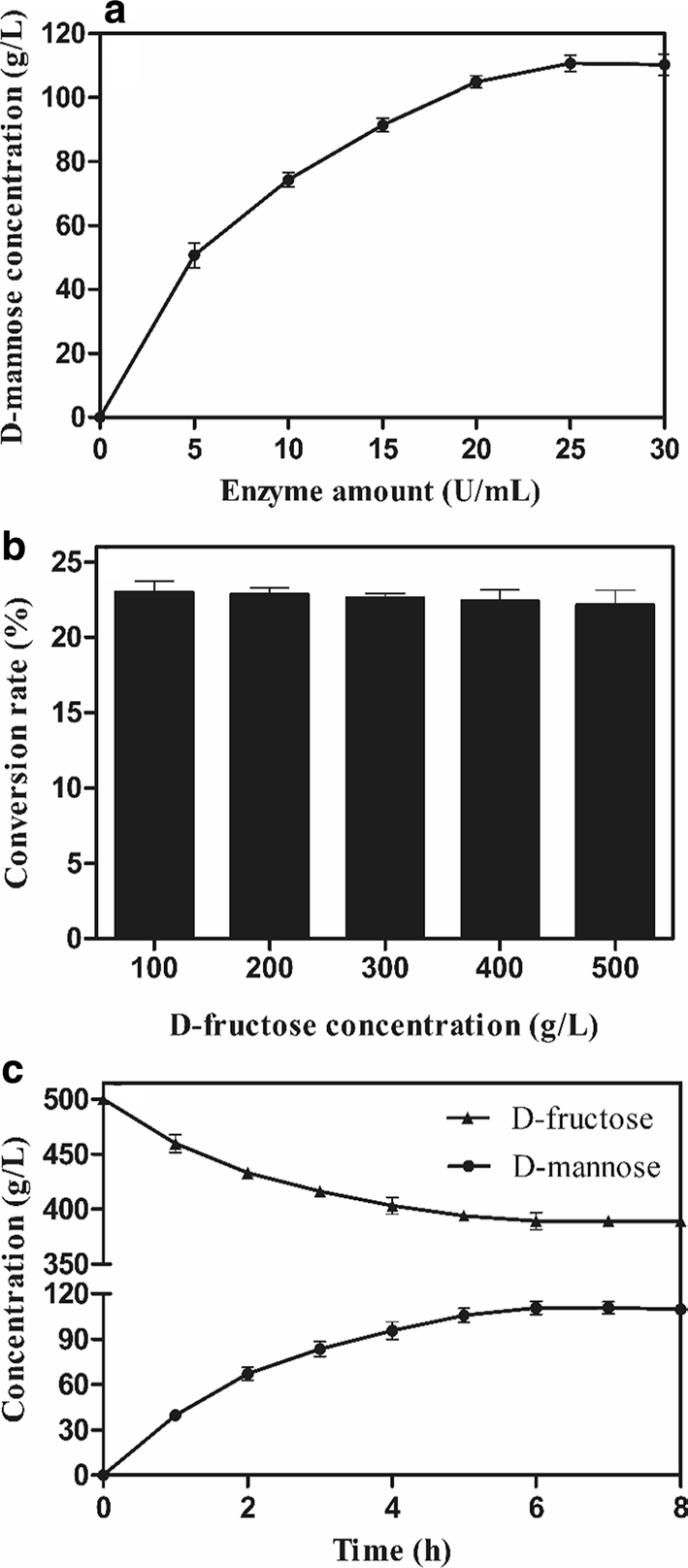
d-Mannose production from d-fructose by recombinant BvLI. **a** Effect of loading of recombinant BvLI on the production of d-mannose. **b** Effect of substrate concentration on the conversion rate of d-mannose. **c** The production of d-mannose from 500 g/L d-fructose by 25 U/mL recombinant BvLI. Data represented the mean ± standard deviation from duplicate experiments

The loading of recombinant BvLI for the d-mannose production from 500 g/L d-fructose were varied from 5 to 30 U/mL. As shown in Fig. 4a, the concentration of d-mannose increased with raising enzyme loading, and the d-mannose concentration reached a plateau at 25 U enzyme/mL. When d-mannose was produced from 100, 200, 300, 400 and 500 g/L d-fructose by 25 U/mL recombinant BvLI (Fig. 4b), the concentration of d-mannose increased with raising the concentration of d-fructose, and the corresponding conversion rates were 23%, 22.85%, 22.64%, 22.45% and 22.15%, respectively. As shown in Fig. 4c, when d-mannose production was proceeded in 500 g/L d-fructose including 25 U/mL recombinant BvLI for 8 h, the reaction reached equilibrium at 6 h and 110.75 g/L d-mannose was obtained. The corresponding productivity of d-mannose was 18.46 g/L/h. These results indicated that high levels of d-mannose could be produced from d-fructose by recombinant BvLI.

### l-Ribose synthesis from l-arabinose by recombinant BlAI and BvLI

**Fig. 5:**
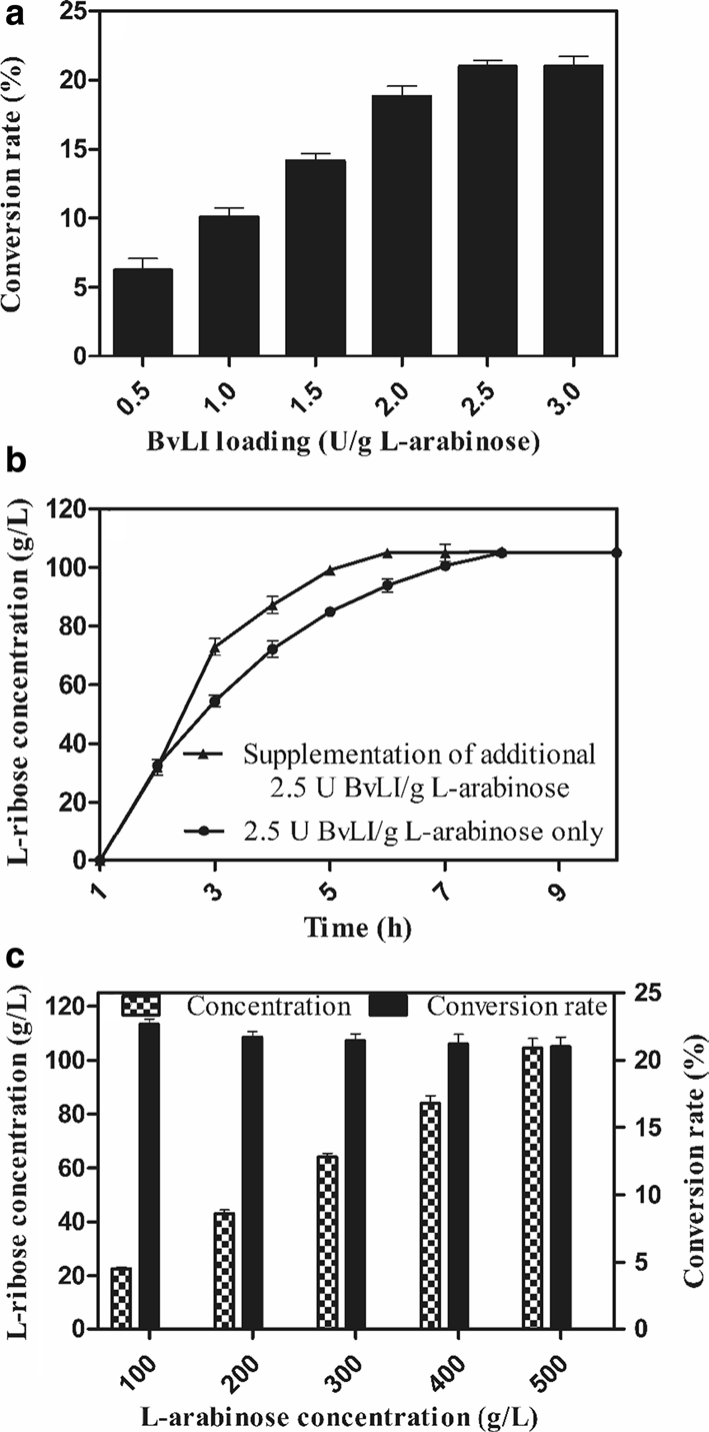
Bioconversion of l-arabinose to l-ribose by the recombinant BlAI and BvLI. **a** The conversion rate of l-ribose with different recombinant BvLI loading. **b** The effect of extra loading of BvLI on the production of l-ribose. **c** Bioconversion of l-ribose using different concentration of l-arabinose. Data represented the mean ± standard deviation from duplicate experiments

The recombinant BlAI displayed maximum activity at pH 7.5 and 50 °C with 1.0 mM Mn^2+^ addition, whereas the maximum activity of recombinant BvLI was observed at 55 °C and pH 6.5, in the presence of 0.1 mM Co^2+^. Therefore, l-ribose synthesis from l-arabinose by applying the recombinant BlAI and BvLI was conducted in an orderly manner. As a result, the conversion rate of l-ribose reached a plateau when recombinant BvLI was 2.5 U/g l-arabinose (Fig. 5a). In addition, in order to know whether the adding extra BvLI has any effect on the conversion, additional 2.5 U/g l-arabinose of BvLI was added at 2 h in the second isomerization stage. It was observed the whole reaction reached faster equilibrium at 6 h, but the final conversion rate of l-ribose and equilibrium ratio of l-arabinose, l-ribulose and l-ribose were not changed compared to that of 2.5 U/g l-arabinose of BvLI at 1 h only (Fig. 5b). Thus, 2.5 U recombinant BvLI was added per gram of l-arabinose in subsequent experiments. When the initial concentrations of l-arabinose were 100, 200, 300, 400 and 500 g/L, respectively, the increase of l-ribose concentration along with increasing l-arabinose concentration. The corresponding conversion rates of l-ribose were 22.7%, 21.7% 21.4%, 21.2% and 21%, respectively, indicating that a similar level of conversion rate could be retained at a high concentration of substrate. And 105 g/L l-ribose were obtained from 500 g/L l-arabinose and leaving 212.42 g/L l-ribulose in 8 h. The corresponding productivity of l-ribose was 13.12 g/L/h and the equilibrium ratio of l-arabinose, l-ribulose and l-ribose was approximately 1.74:2.02:1 (Fig. 5c).

## Discussion

In this study, a putative d-lyxose isomerase from *B. velezensis* was identified and overexpressed in *E. coli* BL21 (DE3) cells. Biochemical properties analysis has confirmed that the gene product BvLI displayed d-LI activity. The recombinant BvLI exhibited the maximum activity at slightly acidic condition (pH 6.5) with good pH stability from pH 5.0–7.0. In general, the production of high-quality sugars required a weakly acidic pH to reduce unwanted by-products in industry (Javad and Harry 1994; Xu et al. 2014). However, only d-LI from *C*. *laevoribosii* RI-39 and d-LI from *T*. *oceani* DSM 16646 showed the highest activity at pH 6.5 among the previously characterized d-LIs (Table 1). Therefore, from the view of pH, the characteristics of BvLI such as a slightly acidic optimum pH, and highly stable in weak acidity, was favorable for industrial application. Thermal stability is an important property for industrial applications of enzymes. It was reported that the half-life times of d-LIs from *Serratia proteamaculans* KCTC 2936 (Park et al. 2010a), *Providencia stuartii* KCTC 2568 (Kwon et al. 2010), and *B. licheniformis* DSM 13 (Patel et al. 2011) were 0.09, 1.4 and 7 h at 50 °C, respectively. By comparison, the recombinant BvLI exhibited significantly higher thermostability at 50 °C than above three d-LIs. However, the half-life time of d-LI from *Dictyoglomus turgidum* DSM 6724 (Choi et al. 2012) was 9.1 h at 60 °C, and the half-life time of d-LI from *T. oceani* DSM 16646 was 5.64 h at 70 °C (Yu et al. 2016), indicating that the thermostability of recombinant BvLI was relatively lower compared to above two d-LIs.

Metal ions play an important role in the catalytic reaction of d-LIs, and all the reported d-LIs have been identified to be metal-dependent enzymes (Table 1). In general, the optimum metal ions for d-LIs activation was Mn^2+^, followed by Co^2+^ and Ni^2+^ at the optimal concentration of 1 mM. In this study, Co^2+^ and Mn^2+^ have a significant activation effect on the activity of d-LI, but unlike many previously reported d-LIs, the recombinant BvLI preferred Co^2+^ as a cofactor at an extreme low concentration requirement (0.1 mM) to activate its catalytic activity. The maximum activation by Co^2+^ was also reported for d-LI from *D*. *turgidum* DSM 6724, however, a relatively higher concentration (0.5 mM) of Co^2+^ was required. This observation suggested that different d-LIs from various sources may have different metal bias and binding affinity, although the amino acids responsible for metal binding are strictly conserved among all the reported d-LIs. Low concentration metal ion requirement is a favorable character for industrial application, hence, these results indicated that recombinant BvLI may be a potential candidate sugar isomerase for high-value sugars synthesis.

d-LIs efficiently catalyzed the reversible isomerization reaction between aldose and ketose sugars. So the isomerization capacity from aldose to ketose also reflects the capacity of the isomerization from ketose to aldose, although the substrate specificity for ketoses was higher than the corresponding aldoses for all characterized d-LIs (Huang et al. 2018b). In this study, only aldose sugars including d-lyxose, d-mannose and l-ribose were used as substrates for biochemical property measurement, because their corresponding ketose products d-xylulose, d-fructose and l-ribulose could be conveniently determined by cysteine carbazole sulfuric acid method. According to the crystal structure analysis (Protein Data Bank accession number: 3KMH) and mutation results for crucial residues in active center of d-LI from *E. coli* O157:H7, the catalytic mechanism of d-LI was proposed to be a cis-enediolate-based mechanism (Van et al. 2010). d-LIs exhibited higher substrate specificity for some aldose such as d-lyxose and d-mannose, where in the C-2 and C-3 hydroxyl groups were oriented to the left side and the C-4 hydroxyl group was oriented to the right side, than l-ribose with the C-2, C-3 and C-4 hydroxyl groups oriented to the left side (Huang et al. 2018b). Similar as other d-LIs, BvLI exhibited the highest activity towards d-lyxose, followed by d-mannose and l-ribose. The *K*_cat_ value for d-lyxose of recombinant BvLI is higher than d-LI from *E. coli* O157:H7, but lower than other previously characterized d-LIs (Additional file 1: Table S1).

In this work, the potential to biosynthesize d-mannose from d-fructose by recombinant BvLI was evaluated in different d-fructose concentrations. It seemed that the conversion rate remained at a similar level irrespective of d-fructose concentrations at a fixed enzyme loading. Recently, several d-LIs have been identified and used to produce d-mannose. For example, the 10% (w/v) d-mannose was obtained from 50% (w/v) d-fructose in 5 h by d-LI from *S*. *proteamaculans* KCTC 2936, with a conversion rate of 20% (Park et al. 2010a). The free d-LI from *P*. *stuartii* KCTC 2568 produced 150 g/L d-mannose from 600 g/L d-fructose in 2 h, giving a conversion rate of 25% and a productivity of 75 g/L/h (Park et al. 2010b). d-LI from *T*. *oceani* DSM 16646 produced 101.6 g/L d-mannose from 400 g/L d-fructose in 9 h, giving a conversion rate of 25.4% and a productivity of 11.29 g/L/h (Yu et al. 2016). d-LI from *Thermoflavimicrobium dichotomicum* produced 110.5 g/L d-mannose from 500 g/L d-fructose with a conversion rate of 25.0% in 6 h (Zhang et al. 2018). By comparison, the conversion rate of d-mannose by recombinant BvLI was higher than the previous reported d-LI from *S*. *proteamaculans* KCTC 2936, but slightly lower than the previous reported d-LI from *P*. *stuartii* KCTC 2568, *T*. *dichotomicum* and *T*. *oceani* DSM16646. It was wealth noted that the production of d-mannose from d-fructose by d-LI from *P*. *stuartii* was carried out in an alkaline condition (pH 7.5). It was considered that alkaline condition might cause browning reactions (Chouayekh et al. 2007; Huang et al. 2018a), which was unfavorable for the production of d-mannose in industry. The recombinant BvLI displayed its maximum activity at a slightly acidic pH (pH6.5), which was similar to d-LI from *T*. *oceani* DSM 16646. Moreover, in this study, the productivity of d-mannose by using recombinant BvLI was 1.64-fold higher than d-LI from *T*. *oceani* DSM 16646 in the optimized condition. These results indicated that the recombinant BvLI possesses good potential to biosynthesize d-mannose from d-fructose.

In this study, the successive biocatalysis of l-arabinose isomerase (BlAI) and d-lyxose isomerase (BvLI) was employed for synthesis of l-ribose from l-arabinose. Increasing BvLI loading resulted in a higher conversion rate as expected, and the equilibrium reached at the loading of 2.5 U/g l-arabinose of BvLI (Fig. 5a). Supplementation of extra BvLI could improve the catalysis speed, but had not benefit for further increase of conversion rate, indicating that the isomerizations are equilibrium processes with thermodynamically limited yields of the products (Delidovich et al. 2017). Generally, for l-ribose synthesis from l-arabinose by a two-step isomerization reaction, the production of l-ribose from l-ribulose was a rate-limiting step in the whole reaction and final conversion rates mostly ranged from 15 to 25% (Kim et al. 2014; Patel et al. 2017). For example, the production of l-ribose from 50 g/L l-arabinose by using l-AI from *Thermoanaerobacterium saccharolyticum* NTOU1 and l-ribose isomerase from *Geodermatophilus obscurus* DSM 43160, giving a conversion rate of 15.9% in 12 h, and the corresponding productivity of l-ribose was 0.66 g/L/h (Hung et al. 2015). When l-AI from s*higella flexneri* and d-LI from *C. laevoribosii* RI-39 were used as biocatalysts in an orderly manner, 2.53 mM l-ribose was obtained from 10 mM l-arabinose after 16 h, giving a conversion rate of 25% and a productivity of 0.024 g/L/h (Patel et al. 2017). In this study, the conversion rate of l-ribose was higher than the study of Hung et al. while the productivity of l-ribose by using recombinant BlAI and BvLI was 19.88-fold and 546.67-fold higher than the study of Hung et al. and Patel et al., respectively. It was reported that xylulose conversion from xylose was significantly improved by removal of xylulose from the reaction mixture using simultaneous isomerization and reactive extraction (Li et al. 2012). The conversion rate of l-ribose might be enhanced by removal of l-ribose to weaken end-product inhibition. Therefore, the separation of l-ribose and reuse of the remaining substrate were significant for industrial production of l-ribose and need to be further investigated in the future. However, it was worth noted that the separation of d-mannose or l-ribose from final reaction mixture was not easy It has been reported that l-ribose could be separated from l-arabinose by simulated moving-bed chromatography (SMBC) (Juza et al. 2000; Song et al. 2019). Thus, SMBC would be a promising technique to separate high-value monosaccharides such as d-mannose and l-ribose from different isomers.

It was also reported that the conversion rate of l-ribose could reach over 70% by a single-step isomerization reaction using d-mannose-6-phosphate isomerase from *Thermus thermophilus* or l-ribose isomerase from *Actinotalea fermentans* ATCC 43279 and l-ribulose as substrate (Yeom et al. 2011; Tseng et al. 2017). However, it should be worth noted that l-ribulose was rare in nature and too expensive to produce l-ribose in industry. Therefore, l-arabinose should be considered as an ideal raw material for the synthesis of l-ribose, and further works still need to be carried out to improve the conversion rate of l-ribose from l-arabinose.

In conclusion, a putative d-lyxose isomerase from *B. velezensis* CICC 24777 was cloned, expressed and characterized. The BvLI exhibited its maximum activity and excellent stability at a weakly acidic environment (pH6.5), and also exhibited a low concentration requirement of Co^2+^, indicating that BvLI is a novel d-LI. The recombinant BvLI demonstrated good potential to synthesize d-mannose from d-fructose. It can also be used to synthesize l-ribose from l-arabinose with l-AI by a two-step isomerization reaction. These results indicated that BvLI may be a good candidate isomerase for production of d-mannose and l-ribose.

## Supplementary information

**Additional file 1: Table S1.** Comparsion of *K*_cat_ of various d-LIs. **Figure S1.** The phylogenetic tree analysis of d-lyxose isomerases of 19 amino acid sequences. (●) For this study. Numbers on nodes represent percentage bootstrap values for 1000 replicates. **Figure. S2** Multiple alignment of the amino acid sequences of BvLI and other d-LIs from various microbiology. **Figure. S3** SDS-PAGE analysis of purified recombinant BlAI. Lane M: protein marker; Lane 1, induced cell debris; Lane 2: crude extract of induced cell lysate; Lane 3: purified recombinant BlAI. **Figure. S4** The effects of Co^2+^ and Mn^2+^ concentration on the activity of recombinant BvLI. Data represented the mean ± standard deviation from triplicate experiments.

## Data Availability

Not applicable.

## Abbreviations

d-LI: d-lyxose isomerase
l-AI: l-arabinose isomerase
BvLI: *Bacillus velezensis* d-lyxose isomerase
BlAI: *Bacillus licheniformis* l-arabinose isomerase
SDS-PAGE: sodium dodecyl sulfate–polyacrylamide gel electrophoresis
IPTG: isopropyl-β-d-thiogalactopyranoside
LB: Luria–Bertani medium
HPLC: high-performance liquid chromatography
