# Supplementary material for: Characterization of a d-lyxose isomerase from Bacillus velezensis and its application for the production of d-mannose and l-ribose
Source: AMB Express. 2019 Sep 16;9:149. doi: 10.1186/s13568-019-0877-3 (PMC6746899; doi:10.1186/s13568-019-0877-3)
Supplement: Supplementary file 1 — Additional file 1: Table S1. Comparsion of Kcat of various d-LIs. Figure S1. The phylogenetic tree analysis of d-lyxose isomerases of 19 amino acid sequences. (●) For this study. Numbers on nodes represent percentage bootstrap values for 1000 replicates. Figure. S2 Multiple alignment of the amino acid sequences of BvLI and other d-LIs from various microbiology. Figure. S3 SDS-PAGE analysis of purified recombinant BlAI. Lane M: protein marker; Lane 1, induced cell debris; Lane 2: crude extract of induced cell lysate; Lane 3: purified recombinant BlAI. Figure. S4 The effects of Co2+ and Mn2+ concentration on the activity of recombinant BvLI. Data represented the mean ± standard deviation from triplicate experiments. [file 13568_2019_877_MOESM1_ESM.docx]

**Additional file** **1:**


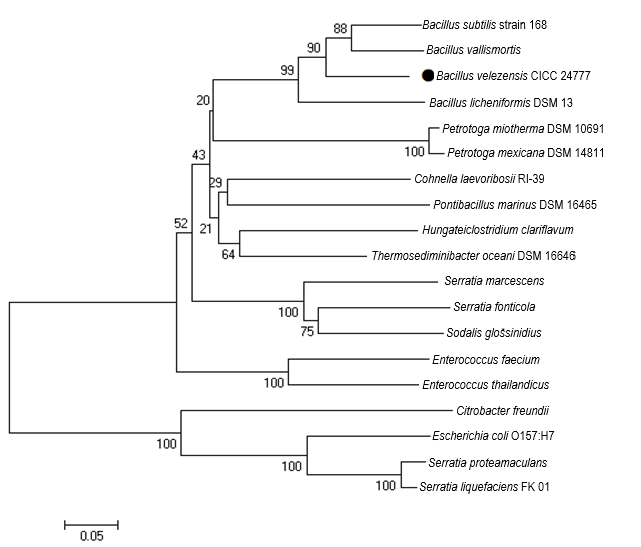


**Fig S1**. The phylogenetic tree analysis of D-lyxose isomerases of 19 amino acid sequences. Numbers on nodes represent percentage bootstrap values for 1000 replicates. *Bacillus subtilis* strain 168 D-LI (GenBank ID: AIY91703); *Bacillus vallismortis* D-LI (NCBI reference sequence: WP_010330845.1); *Bacillus licheniformis* DSM 13 D-LI (GenBank ID: AAU22106.1); *Petrotoga miotherma* DSM 10691 D-LI (GenBank ID: PNS01248.1); *Petrotoga mexicana* DSM 14811 D-LI (GenBank ID: PNR99621.1); *Cohnella laevoribosii* RI-39 D-LI (GenBank ID: ABI93960.1); *Pontibacillus marinus* DSM 16465 D-LI (GenBank ID: KGX84558.1); *Hungateiclostridium clariflavum* D-LI: (NCBI reference sequence: WP_014255395.1); *Thermosediminibacter oceani* DSM 16646 D-LI (GenBank ID: ADL08607.1); *Serratia marcescens* D-LI (GenBank ID: BBG69721.1); *Serratia fonticola* D-LI (NCBI reference sequence: WP_021178408.1); *Sodalis glossinidius* D-LI (NCBI reference sequence: WP_011410362.1); *Enterococcus faecium* D-LI (GenBank ID: SJX71758.1); *Enterococcus thailandicus* D-LI (GenBank ID: OJG94896.1); *Citrobacter freundii* D-LI (GenBank ID: GCB41955.1); *Escherichia coli* O157:H7 D-LI (GenBank ID: Q8X5Q7); *Serratia proteamaculans* D-LI (GenBank ID: BAJ07463.1); *Serratia liquefaciens* FK 01 D-LI (GenBank ID: GAK25190.1). ● For this study.


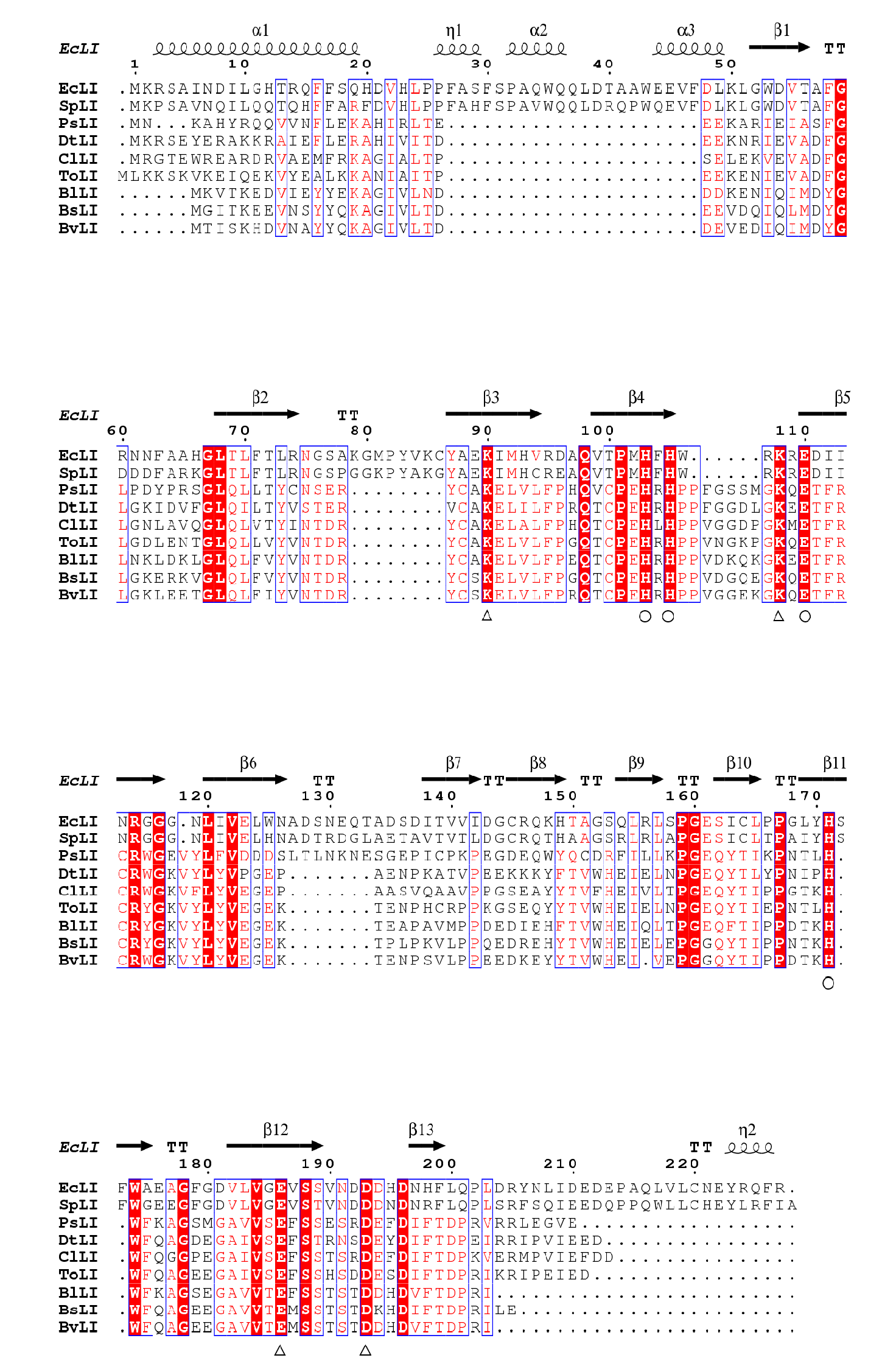


**Fig. S2** Multiple alignment of the amino acid sequences of BvLI and other D-LIs from various microbiology. According to complex structure of *E. coli* D-LI bound to D-fructose (PDB no. 3MPB), (△) the residues involved in the substrate binding sites; (○) the residues involved in the metal coordination and substrate binding sites. ECLI: *E. coli* O157:H7 D-LI (GenBanK ID: Q8X5Q7); SpLI: *Serratia proteamaculans* KCTC 2936 D-LI (GenBanK ID: BAJ07463.1); PsLI: *Providencia stuartii* KCTC 2568 D-LI (GenBanK ID: EDU58657); DtLI: *Dictyoglomus turgidum* DSM 6724 D-LI (GenBanK ID: YP_002352606.1); ClLI: *Cohnella laevoribosii* RI-39 D-LI (GenBanK ID: ABI93960.1); ToLI: *Thermosediminibacter oceani* DSM 16646 D-LI (GenBanK ID: ADL08607.1); BlLI: *Bacillus licheniformis* DSM 13 D-LI (GenBanK ID: AAU22106.1); BsLI: *Bacillus subtilis* strain 168 D-LI (GenBanK ID: AIY91703); BvLI: *Bacillus velezensis* D-LI (GenBanK ID: MK836420). The alignment was performed by using ESPript (<http://espript.ibcp.fr/ESPript/ESPript/>).


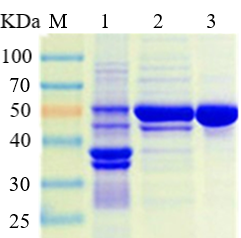


**Fig. S3** SDS-PAGE analysis of purified recombinant BlAI. Lane M: protein marker; Lane 1, induced cell debris; Lane 2: crude extract of induced cell lysate; Lane 3: purified recombinant BlAI.


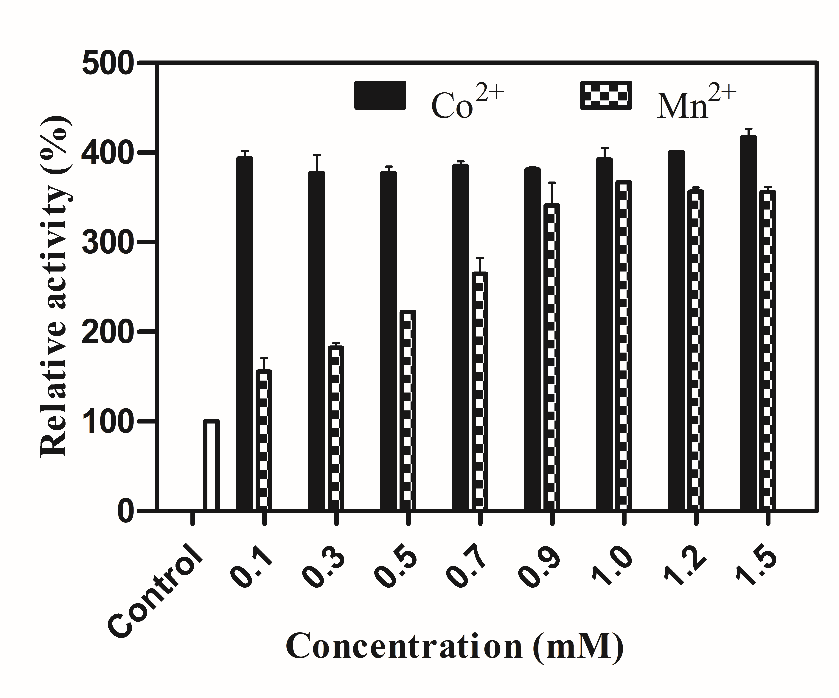


**Fig. S4** The effects of Co^2+^ and Mn^2+^ concentration on the activity of recombinant BvLI. Data represent the mean±standard deviation from triplicate experiments.

**Table S1.** Comparsion of *K*_cat_ of various D-LIs.

| Origins | *K*_cat_ (min^-1^)  D-lyxose D-mannose L-ribose | | | References |
| --- | --- | --- | --- | --- |
| *Cohnella laevoribosii*  RI-39 | 114,120 | 2766 | 1584 | Cho et al. ([2007](https://doi.org/10.1128/JB.01568-06)) |
| *Providencia stuartii*  KCTC 2568 | 1,880,400 | 158,400 | NR | Kwon et al. ([2010](https://doi.org/10.1016/j.jbiosc.2009.12.011)) |
| *Serratia proteamaculans* KCTC 2936 | 1,848,600 | 970,200 | NR | Park et al. ([2010a](https://doi.org/10.1111/j.1472-765X.2010.02903.x)) |
| *Escherichia coli* O157:H7 | 822 | 762 | NR | Van et al. ([2010](https://doi.org/10.1016/j.jmb.2010.06.063)) |
| *Bacillus licheniformis* DSM 13 | 5880 | 2580 | NR | Patel et al. ([2011](http://dx.doi.org/10.1128/AEM.02693-10)) |
| *Dictyoglomus turgidum*  DSM 6724 | 3570 | 178 | NR | Choi et al. ([2012](https://doi.org/10.1007/s10529-012-0874-y)) |
| *Thermosedimini*-  *bacter oceani* DSM 16646 | 3108 | 5686 | NR | Yu et al. ([2016](https://doi.org/10.1016/j.procbio.2016.08.023)) |
| *Bacillus velezensis* CICC 24777 | 1335.89 | 1213.46 | 975.45 | This study |

*NR* Not reported
